# Long-range correlations in the mechanics of small DNA circles under topological stress revealed by multi-scale simulation

**DOI:** 10.1093/nar/gkw815

**Published:** 2016-09-22

**Authors:** Thana Sutthibutpong, Christian Matek, Craig Benham, Gabriel G. Slade, Agnes Noy, Charles Laughton, Jonathan P. K. Doye, Ard A. Louis, Sarah A. Harris

**Affiliations:** 1 School of Physics and Astronomy, University of Leeds, Woodhouse Lane, Leeds LS2 9JT, UK; 2 Theoretical and Computational Science Center (TaCS), Science Laboratory Building, Faculty of Science, King Mongkut's University of Technology Thonburi (KMUTT), 126 Pracha-Uthit Road, Bang Mod, Thrung Khru, Bangkok 10140, Thailand; 3 Rudolf Peierls Centre for Theoretical Physics, University of Oxford, Parks Road, Oxford OX1 3PU, UK; 4 UC Davis Genome Centre, Health Sciences Drive, Davis, CA 95616, USA; 5 Department of Physics, São Paulo State University, Rua Cristovão, São José do Rio Preto, SP 15054-000, Brazil; 6 Department of Physics, Biological Physical Sciences Institute, University of York, York, YO10 5DD, UK; 7 School of Pharmacy and Centre for Biomolecular Sciences, University of Nottingham, University Park, Nottingham NG7 2RD, UK; 8 Physical and Theoretical Chemistry Laboratory, Department of Chemistry, University of Oxford, South Parks Road, Oxford OX1 3QZ, UK; 9 Astbury Centre for Structural and Molecular Biology, University of Leeds, Woodhouse Lane, Leeds LS2 9JT, UK

## Abstract

It is well established that gene regulation can be achieved through activator and repressor proteins that bind to DNA and switch particular genes on or off, and that complex metabolic networks determine the levels of transcription of a given gene at a given time. Using three complementary computational techniques to study the sequence-dependence of DNA denaturation within DNA minicircles, we have observed that whenever the ends of the DNA are constrained, information can be transferred over long distances directly by the transmission of mechanical stress through the DNA itself, without any requirement for external signalling factors. Our models combine atomistic molecular dynamics (MD) with coarse-grained simulations and statistical mechanical calculations to span three distinct spatial resolutions and timescale regimes. While they give a consensus view of the non-locality of sequence-dependent denaturation in highly bent and supercoiled DNA loops, each also reveals a unique aspect of long-range informational transfer that occurs as a result of restraining the DNA within the closed loop of the minicircles.

## INTRODUCTION

Compartmentalization is well established as a strategy that has evolved to manage the complex environment of the cell. Organelles, vesicles and proteinaceous microcompartments in bacteria all provide physical boundaries that enable multiple metabolic processes to run concurrently and which optimize the speed of transfer of biological information through modified diffusion distances. Similarly, chromosome capture techniques have now revealed that genomes are partitioned into independent topological domains (1). While the physical nature of these genomic boundaries is currently poorly understood, whenever the anchor point is sufficiently robust then the DNA will be partitioned into a series of mechanically coupled elements which are physically isolated from each other. Here, we have used a combination of statistical physics and multi-scale structural modelling applied to DNA minicircles containing ∼100 base pairs to demonstrate that the mechanics of topologically restrained DNA is determined globally and interactively, not locally. Specifically, we predict the global shape of the minicircles, the probability of defect formation, and how this depends on DNA sequence. We show that there are a variety of mechanisms for long-range information transfer and non-locality through mechanical stress in closed DNA loops, which apply more generally whenever DNA is topologically restrained, such as in genomic DNA. The focus here is on minicircles because DNA loops of this size permit detailed modelling at a range of structural resolutions (atomistic, single base and using phenomenological models), and reveal the interplay between torsional and bending stress within the duplex, which is representative of the most extreme mechanical distortions experienced by supercoiled DNA.

DNA packaging and transcription subject the duplex to static and dynamic mechanical stresses, respectively. In bacteria, DNA is compacted into plectonemic supercoils through under-winding by DNA gyrase (2). The dynamic interplay between the accumulation of positive supercoils due to the passage of RNA polymerase along duplex DNA and the relief of torsional stress by gyrase has been implicated in gene expression heterogeneity and transcriptional bursting in *Escherichia coli* (3). A genome wide analysis of the propensity of the *E. coli* chromosome to denature as a result of superhelical stress has shown that susceptible regions are statistically particularly likely to contain promoter sites, whereas intergenic regions are more robust (4). While supercoiling in eukaryotes is largely stored within nucleosomes, dynamic supercoiling due to transcription has been detected ∼1.4 kb upstream of transcription state sites in active genes (5). Negative supercoiling promotes the formation of single-stranded regions by weakening base stacking. This has implications for gene regulation by direct control over the formation of the open complex during transcription, which is facilitated by weaker base stacking. Supercoiling can also affect DNA recognition by activator and repressor proteins through changes in the widths of the major and minor grooves (6), and most dramatically by promoting non-canonical DNA structures such as cruciform or quadruplexes (7,8), which can absorb the superhelical stress. DNA melting and reannealing into non-canonical conformations is implicated in the regulation of the c-myc proto-oncogene by the Far Upstream Sequence Element (FUSE), which how been shown to melt into a single stranded region in response to negative supercoiling during transcription (9). Other examples include the ilvP_G_ promoter in *E. coli*, which is activated by the binding of IHF to a distant site that has a high propensity to denature when subjected to superhelical stress. Protein binding reanneals the duplex, which then causes the superhelical stress to be transmitted to the next most susceptible site upstream, initiating transcription (10). The formation of non-canonical DNA by repetitive sequences, in which slipped base pairing occurs frequently as it relieves torsional stress within the duplex, has received particular attention due to the implication for human disease (11).

The most severe mechanical distortion in plectonemic DNA occurs at the apices, where the DNA is forced to be both bent and supercoiled (12,13). Bending stress also occurs during the formation of protein-constrained loops, which are involved in cellular processes including transcription, replication and recombination and which allow distal regions of DNA to contact and affect each other (14,15), and which may respond to supercoiling in a complex sequence-dependent manner (16). Intriguingly, the sensitivity of protein mediated looping by the λ repressor to DNA supercoiling in *E. coli* has been suggested to provide a sensor for the energy levels present in the cell (17), since there is a direct connection between ATP levels and gyrase activity (18). Sufficient levels of bending stress can also generate kink defects in the DNA, in which the stacking interactions between successive base steps are broken (19–22). Kinks can be found within the nucleosome (23,24) and in CAP–DNA complexes (25) and are highly sequence dependent. Extrachromosomal microDNA circles, which contain between 200 and 400 bp, have been identified in mammalian cells, but seem to result from faulty or incomplete DNA replication or repair rather than imparting biological function (26). The prevalence of these microDNAs suggests that the stability of the genome may depend on the detailed response of individual sequences to severe bending stress, as their appearance will depend critically on the physical attributes of the DNA fragments involved.

Synthetic DNA minicircles, which contain between 60 and 500 bp, have proven particularly useful as probes of the mechanical transitions that occur due to extreme bending and torsional stress. The minicircle size and supercoiling can be precisely controlled, the partitioning between twist and writhe can be studied either with gel electrophoresis or by imaging with AFM (27) or cryo-EM (28) and the presence of single stranded regions can be detected enzymatically (22,29–31). However, since none of these experimental techniques are able to provide detailed structural information for the stressed DNA loops, we have used computer simulation to compare the denaturation of small circles for three different sequences, to visualize the resulting structural defects within the minicircles, and to make predictions of the positions of these defects within the circles that should be experimentally testable given modest improvements in the biochemical methods available.

Three complementary computational modelling techniques have been used to determine the sequence dependence of supercoiling induced denaturation in these DNA minicircles. At the highest resolution, we have performed fully atomistic MD simulations of the DNA surrounded by sufficient Na^+^ and Cl^−^ ions to be equivalent to 100mM ionic strength and solvated by explicit water molecules. At intermediate resolution, we have used the coarse-grained oxDNA model (32,33), which represents the DNA at the level of individual nucleotide bases and represents the solvent implicitly through the effective interaction potentials. Due to its coarse-grained nature, oxDNA simulations can access much longer time scales (70 μs) than atomistic MD simulations (50 ns). Importantly for the current application, oxDNA has been shown to accurately model the response of DNA to strong torsional and bending stress (8,12,34). To explore denaturation without the limitations in size or time scale inherent in computer simulations, we have also modelled strand separation by negative superhelicity as a two-state transition using equilibrium statistical mechanics (35,36) as implemented in the SIDD (Stress Induced Duplex Destablization) algorithm. All three of these complementary theoretical techniques have revealed unique aspects of long-range informational transfer that occur as a result of restraining the DNA ends within the closed loop of the minicircles.

## MATERIALS AND METHODS

### Sequences investigated

**Figure 1.**
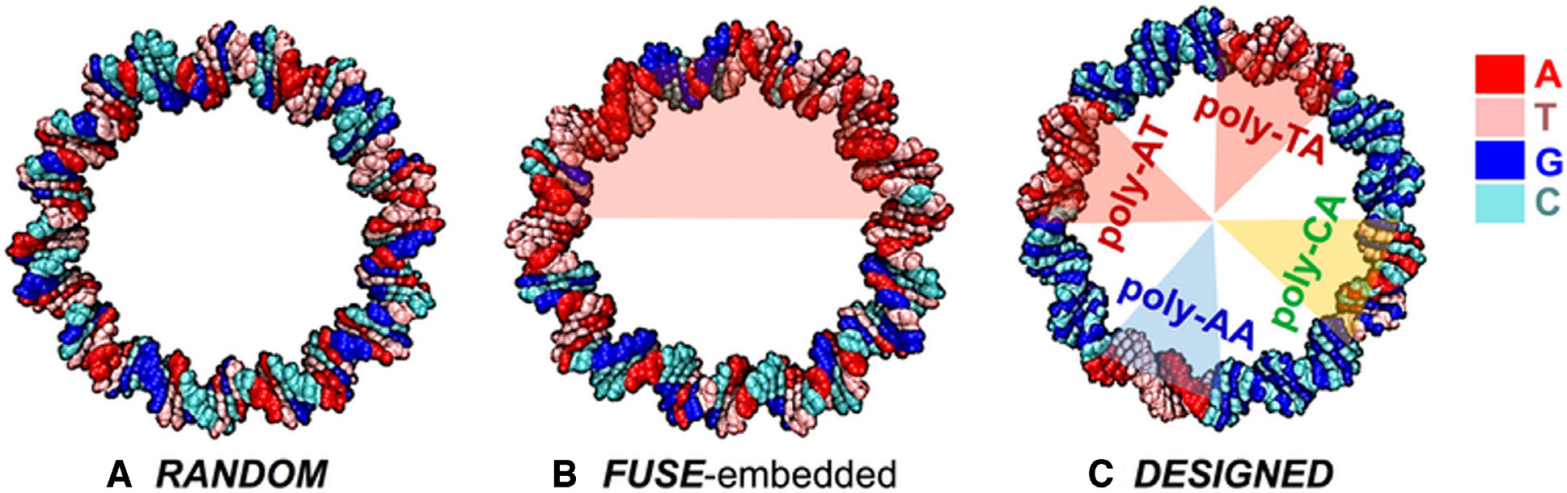
(**A**) ***RANDOM*:** sequence containing approximately equal AT and GC base pairs, and used in the biochemical study of Du *et al*.; (**B**) ***FUSE*:** sequence obtained by embedding an A+T-rich Far Upstream Sequence Element (FUSE) into the *RANDOM* sequence; (**C**) ***DESIGNED*:** sequence containing repetitive sequence motifs. The coloured inserts indicate the FUSE element (pink, centre) in *FUSE* sequence, and poly-TA and poly-AT (pink, right), poly-AA (blue, right), and poly-CA (yellow, right) elements in *DESIGNED* sequence, respectively.

Three sequences were chosen for investigation, and are provided in full in the Supplementary Information. The RANDOM sequence contained approximately equal AT and GC base pairs (see Figure 1A). This sequence has been investigated experimentally by Du *et al*. (22), and by previous atomistic MD simulations (37). In the second, a 50-bp section of the RANDOM sequence is replaced by a 50-bp section of the AT-rich biologically active FUSE element (9), resulting in a circle containing an AT-rich section and a segment of random sequence (see Figure 1B). The third is a rationally designed DNA sequence containing repetitive elements, in which four sequence motifs of mechanical interest have been embedded into a GC only circle. The DESIGNED sequence contains eight regions in which the four 15 bp poly-CG regions alternate with the other four 12 bp sequence motifs: poly-TA, poly-AT (both in pink), poly-AA (blue), and poly-CA (yellow) (see Figure 1C).

The superhelical stress in the DNA is determined by the superhelical density σ:

\begin{equation*}\sigma = \frac{{Lk - L{k_0}}}{{L{k_0}}}\end{equation*}

where *Lk* is the number of helical turns, *Lk*_0_ is the number of helical turns in a relaxed DNA molecule and Δ*Lk = Lk – Lk_0_* is the number of helical turns away from a relaxed helical state, which is enforced by the topological constraint. The number of turns in a relaxed molecule, *Lk_0_*, has a complicated dependence on DNA sequence, temperature, and salt concentration. In the simulations, it also depends on the precise modelling method that is being employed. The version of the AMBER forcefield used for the atomistic MD calculations (see below) underestimates the twist of DNA by around 0.7°, whereas for oxDNA the pitch is around 10.36 base pairs, which is also a bit less than the experimental value. While these discrepancies are small for sequences containing only ten or twenty base pairs, they must be corrected in simulations of DNA circles containing in excess of 100 bp, or the superhelical density (and hence the mechanical stress) would not be directly comparable with results obtained with oxDNA and SIDD. We estimated that two base pairs should to be added into the atomistic DNA minicircles, resulting in circles containing 102 base pairs for Δ*Lk* ∼ −0.5 (compared to 100 for oxDNA and SIDD) and 108 base pairs for Δ*Lk* ∼ 0 and Δ*Lk* ∼ −1 (compared to 106 for oxDNA and SIDD).

While oxDNA and SIDD both do not have sequence dependent twist parameters, the twist of base steps within AMBER is highly sequence dependent, consequently, the overall twist and therefore the superhelical density can be difficult to assign precisely, as shown in Table 1, and discussed in the Results section. Consequently, all Δ*Lk* values quoted are approximate due to the uncertainty in assigning *Lk_0_*. In building the models, we assumed that all of the negatively supercoiled minicircles have *Lk* = 9 and the torsionally relaxed minicircles have *Lk* = 10.

**Table 1.** Factors influencing the base pair breathing rates observed in atomistic MD simulations of RANDOM, FUSE-embedded and DESIGNED minicircles at Δ*Lk* ≈ −0.5: sequence dependent superhelical density; average twist deviation calculated from atomistic MD simulations of linear sequences; TA and CA basepair step contents and the mechanical stress associated with deformations away from the average (see Supplementary Table S6), as calculated from diagonal elements of the stiffness matrix (see Supplementary Table S5). Darker shading indicates a stronger influence on DNA breathing

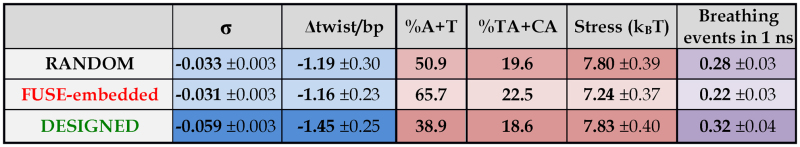						

### Statistical mechanical calculations

SIDD calculations were performed on the three sequences of the DNA mini-circles at superhelical densities σ = Δ*Lk/Lk_o_* ≈ −0.05 and −0.1. Copolymer energetics, which assigns a single value to the strength of all AT base pairs and a single (but different) value to all GC base pairs was used. The temperature was set to be 300 K, and the ionic strength was 0.1 M NaCl. The SIDD method was used to calculate the equilibrium probability of denaturation of each base pair in each sequence. SIDD provides the relative denaturation probabilities for all base pairs in a given sequence, but not the absolute probabilities. Consequently, the probabilities (shown in Supplementary Figure S2) have been normalized separately for each minicircle, to enable direct comparison with oxDNA results.

### Coarse-grained simulations with oxDNA

For each sequence and minicircle size, five independent dynamical simulations were run for a simulated time of at least 70 μs each. As oxDNA is an implicit-solvent model, random forces need to be added to Newton's equations to generate the appropriate diffusive motion of particles in solution. In the present work, this was achieved using an Andersen-like thermostat (38) at *T* = 300 K which has been successfully implemented in previous oxDNA calculations (39).

### Atomistic molecular dynamics simulations of DNA minicircles

All simulations were run in 0.1 M NaCl at 300K using the AMBER ff99parmbsc0 forcefield with a Chi-dihedral angle correction (40). Further details of the simulations protocols are provided as supplementary information (see Supplementary Table S1). Atomistic MD runs were performed with GROMACS using 32 processors of the Advanced Research Computing (ARC1 and ARC2) supercomputers, the N8 High Performance Computing cluster and XSEDE Stampede supercomputing resources. A typical simulation cell contained about 450 000 atoms (including water), and required one day for approximately 1.25 ns of MD from ARC1/2 or approximately 35 ns of MD from XSEDE Stampede.

Nine simulations were performed for each of the RANDOM and FUSE sequences considering three different starting register angles: A, B and C (see Supplementary Table S2). Register A had the minor groove of the first base pair step in the DNA sequence bent towards the centre of the circle, while in registers B and C, the positions of DNA base pairs were shifted by +5 and −5, so that three different bending directions for each DNA segment were sampled. To minimize potential artefacts introduced by our choice of a perfectly circular starting structure, all minicircles were subjected to an additional equilibration period (typically 1 ns) in which just the hydrogen bonds were restrained. The resulting starting structure was only accepted if no denaturation events were observed during the first 1 ns of fully unrestrained MD. Simulations were typically run for 50 ns; for the higher superhelical density Δ*Lk* ≈ −1, the simulations were continued until a structural disruption was observed that persisted for longer than 10 ns. For the DESIGNED sequence, we performed 45 simulations, 15 for each register angle. We ran all the DESIGNED simulations for at least 20ns. Further details of the simulations performed are provided in Supplementary Tables S2 and S3 in the supplementary information.

### Analysis of structural disruptions in the atomistic MD and coarse-grained simulations

In the coarse-grained oxDNA simulations, a DNA base pair was counted as open when the hydrogen-bond interaction potential in oxDNA was <10% of the depth of the effective potential (corresponding to a hydrogen-bond energy of less than −3.03 × 10^−21^J). The denaturation probability profiles were calculated from the results of all five independent runs; equilibration and sampling were assessed by checking that the calculated denaturation probabilities are robust against removal of one of the data sets from the statistical averaging.

In the atomistic MD simulations, the middle hydrogen bond distances were measured between the N1 atom of adenine and the hydrogen atom associated with the N3 atom of thymine for an AT base pair, and between the N3 atom of Cytosine and the hydrogen atom associated with the N1 atom of Guanine for a GC base pair (see Supplementary Figure S1) using the PTRAJ module in AMBER. A base pair is classified as disrupted when its middle hydrogen bond distance exceeds 4 Å and a reversible breathing event was counted whenever a base pair opened and then closed within 1 ns. The error in the breathing frequency was calculated from the standard deviation observed in the nine simulations of each sequence.

### Calculation of the helical parameters from atomistic MD of linear sequences

To obtain the equilibrium twist and helical parameters required to calculate the stiffness matrix for the ff99bsc0χOL4 forcefield, we performed an additional series of 50 ns atomistic MD simulations for 16 sequences of 43-bp linear DNA oligomers (for details of the explicitly solvated simulation protocols see Supplementary Information Table S1). The helical parameters obtained are provided as supplementary information in Supplementary Table S4.

### Calculations of the elastic energy from the stiffness matrix

The elastic energy stored was calculated using the stiffness matrix method, as described by Lankas *et al*. (41). The stiffness matrix is defined as the inverse of the correlation matrix of the six base pair step parameters (*C*):

\begin{equation*}A = {k_B}T{C^{ - 1}}\end{equation*}

\begin{equation*}A = \left[ {\begin{array}{@{}*{6}{l}@{}} {{\boldsymbol{a}_{\boldsymbol{shift}}}}&{{a_{sh - sl}}}&{{a_{sh - ri}}}&{{a_{sh - ti}}}&{{a_{sh - ro}}}&{{a_{sh - tw}}}\\ {{a_{sl - sh}}}&{{\boldsymbol{a}_{\boldsymbol{slide}}}}&{{a_{sl - ri}}}&{{a_{sl - ti}}}&{{a_{sl - ro}}}&{{a_{sl - tw}}}\\ {{a_{ri - sh}}}&{{a_{ri - sl}}}&{{\boldsymbol{a}_{\boldsymbol{rise}}}}&{{a_{ri - ti}}}&{{a_{ri - ro}}}&{{a_{ri - tw}}}\\ {{a_{ti - sh}}}&{{a_{ti - sl}}}&{{a_{ti - ri}}}&{{\boldsymbol{a}_{\boldsymbol{tilt}}}}&{{a_{ti - ro}}}&{{a_{ti - tw}}}\\ {{a_{ro - sh}}}&{{a_{ro - sl}}}&{{a_{ro - ri}}}&{{a_{ro - ti}}}&{{\boldsymbol{a}_{\boldsymbol{roll}}}}&{{a_{ro - tw}}}\\ {{a_{tw - sh}}}&{{a_{tw - sl}}}&{{a_{tw - ri}}}&{{a_{tw - ti}}}&{{a_{tw - ro}}}&{{\boldsymbol{a}_{\boldsymbol{twist}}}} \end{array}} \right]\end{equation*}

The diagonal elements are the force constants of the base pair steps in all six degrees of freedom and the non-diagonal elements describe the coupling between each pair of parameters. The elastic energy of each base pair step can then be obtained by

\begin{equation*}E = \frac{1}{2}\sum\nolimits_{i = 1}^6 {\sum\nolimits_{j = 1}^6 {{a_{ij}}\Delta {p_i}\Delta {p_j},} } \end{equation*}

when ${a_{ij}}$ is an element in the stiffness matrix *A* and $\Delta {p_i}$ is the deviation of base pair step parameter *i* from the average value. Average values, standard deviations and stiffness matrices for each base pair step type derived from simulations of a 43-bp linear DNA oligomer are provided in Supplementary Table S5.

## RESULTS

### Sequence dependent denaturation from SIDD and oxDNA

**Figure 2.**
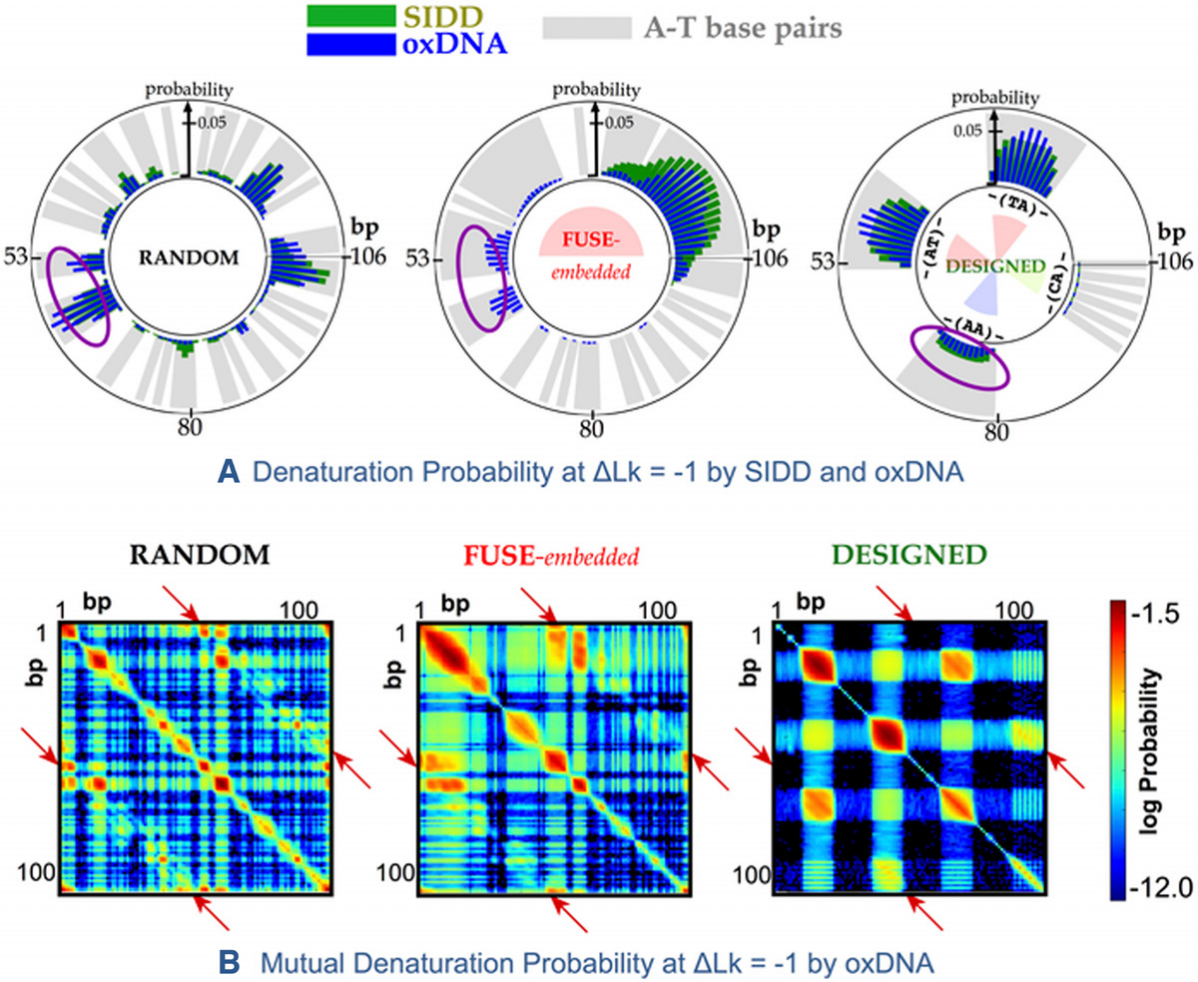
(**A**) Sequence-dependent denaturation probability observed in negatively supercoiled DNA minicircles at Δ*Lk* ≈ −1 of the ***RANDOM, FUSE***-embedded, and ***DESIGNED*** sequences. This probability was calculated using the SIDD model (green) and oxDNA simulation (blue). A-T base pairs are shaded in grey. Secondary denaturation sites due to the cooperative long-ranged kinking predicted by oxDNA simulations are circled. (**B**) 2D colour plots of the log of the probability for a given pair of base pairs to be simultaneously denatured in Δ*Lk* ≈ −1 minicircles, calculated with oxDNA for the same three sequences. Unsampled states are coloured black. Denaturation is enhanced at specific sites separated by ∼180° (e.g. situated antipodally) along the minicircle, as demonstrated by the off-diagonal peaks in the mutual denaturation probability profile (indicated with red arrows).

Figure 2A shows a comparison between the sequence dependence of the base-pair opening probabilities at Δ*Lk* ≈ −1 calculated with SIDD (green) and simulations performed with the oxDNA model (blue). SIDD and the coarse-grained oxDNA calculations agree that single-stranded regions occur with highest probability in the centre of AT-rich regions (shaded in grey) due to the lower thermodynamic stability of AT relative to GC base pairs and the nucleation barrier associated with the loss of base stacking interactions at the interface between ss and dsDNA. Alternating purine-pyrimindine (e.g TA) steps are more susceptible to denaturation because they have reduced base stacking compared to homopolymeric sequences (e.g. AA), consequently in the designed sequence the denaturation probability for bp14-25 (-(TA)-) and bp41-52 (-(AT)-) is enhanced relative to bp68-79 (-(AA)-) according to both oxDNA and SIDD. Comparing the SIDD denaturation profiles of the RANDOM and FUSE-embedded minicircles shows that in this model, substituting one half of the minicircle (1–50 bp) with the more AT-rich FUSE sequence switches the most probable denaturation site from 105 bp (in the RANDOM sequence) to bp9 (in the FUSE containing circle). The inclusion of the denaturation prone AT-rich FUSE element reduces the probability of a base pair breaking within the more robust sections of the minicircle by acting as a sink for the superhelical stress. Defect formation is associated with a reduction in the superhelical stress throughout the whole topologically constrained loop, and therefore has a global effect on the melting probability. Moreover, the nucleation barrier, which favours single over multiple bubbles, alters the relative denaturation probability for the FUSE and RANDOM minicircles in the region spanning bp1 within the SIDD model, because replacement by the FUSE element introduces a long stretch of denaturation prone AT-rich DNA in which a large bubble can form. The non-locality of supercoiling induced melting due to long-range communication of changes in superhelical stress throughout a given topological domain is not specific to small DNA circles or to these particular sequences, but may be expected to occur whenever mechanical constraints are imposed on the ends of a section of DNA.

### Bending stress is relieved by co-operative kinking in oxDNA models

**Figure 3.**
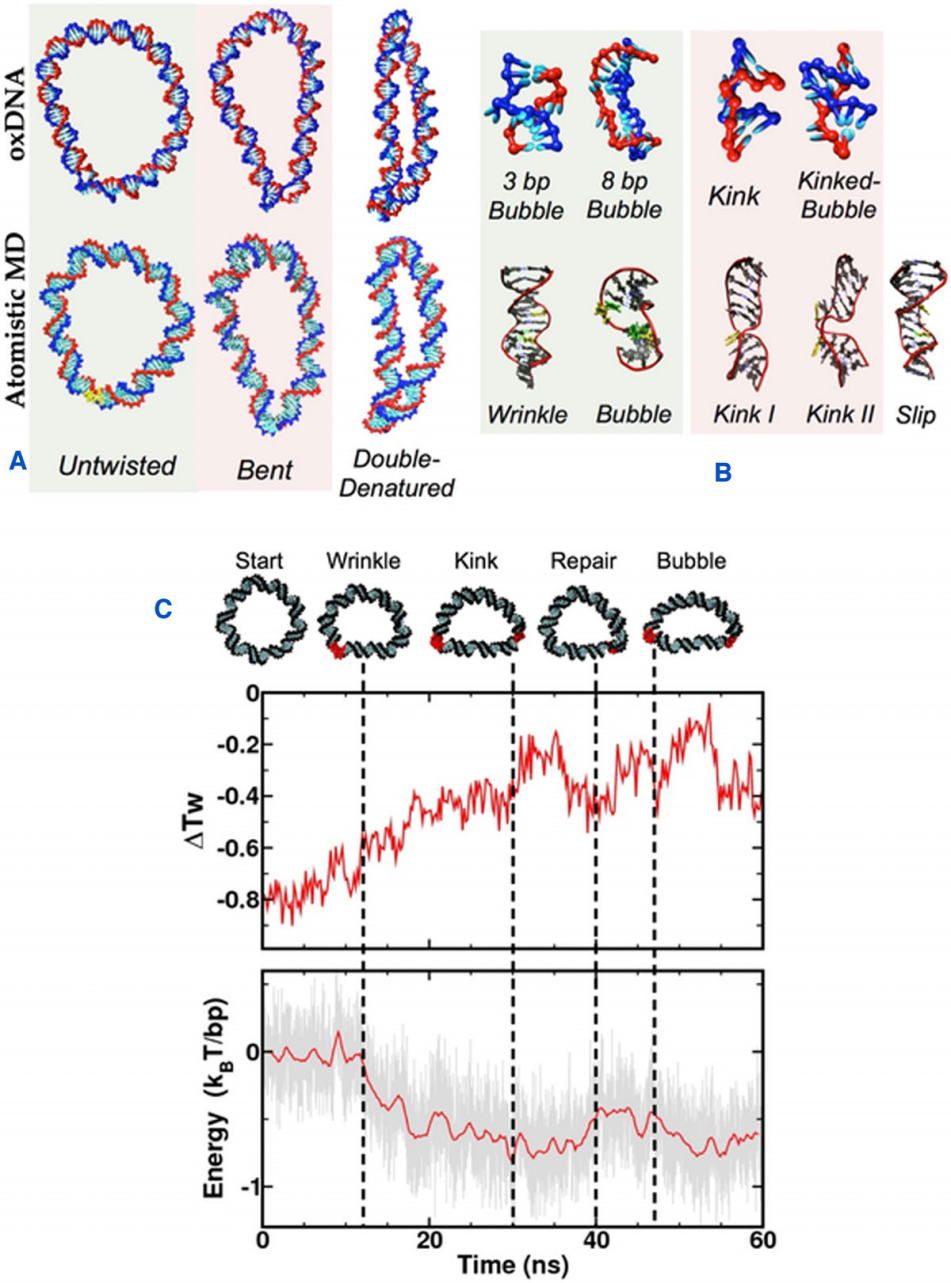
(**A**) Representative global conformations of DNA minicircles containing defects observed at Δ*Lk* ≈ −1 in oxDNA (top) and atomistic MD (bottom). (**B**) Detailed structures of the defects observed in the oxDNA and atomistic MD simulations (where yellow indicates a denatured site). (**C**) An example of the choreography of cooperative denaturation observed in a Δ*Lk* ≈ −1 RANDOM minicircle in a representative atomistic MD simulation: (top) Snapshots of the disrupted minicircles showing the time dependent structural changes (middle) Time evolution of the average twist deviation showing the relaxation of the superhelical stress due to these structural disruptions (bottom) Time evolution of the reduction in the stress energy relative to the starting structure (which is arbitrarily set to be zero) during this atomistic MD simulation. Dashed lines indicate which DNA structures emerge as a result of the twist and energetic relaxation during the atomistic MD trajectory.

Previous calculations of the SIDD profile of the FUSE sequence agreed quantitatively with the experimental data when analysed in the context of a 3.5 kb genomic sequence (42). The SIDD model correctly predicted that the FUSE element melts bimodally between superhelical densities σ = −0.04 and σ = −0.06 at the exact sites that correspond to the early and late melting regions. In DNA minicircles, the general features of the denaturation profiles obtained are broadly similar for the SIDD and oxDNA models. However, differences arise due to the precise geometry of the circle and the tight bending within the loop, as these are not considered by the SIDD method. Denaturation in the oxDNA model can relieve bending as well as torsional stress by the formation of a kink in the denatured section that localizes the bending to this more flexible region. The mutual denaturation probability for pairs of DNA bases within the Δ*Lk* ≈ −1 minicircle observed in the oxDNA simulations, particularly in the FUSE-embedded sequence (see Figure 2A (purple circles), Figure 2B and Supplementary Figure S2) shows a co-operative enhancement in the likelihood of defect formation for pairs of bases located at opposite positions within the DNA minicircle, as this releases the maximum amount of bending stress. This mechanics is not captured by SIDD, as bending stress within the minicircle is not included in this model. Unlike in simple linear DNA fragments where the effect of any structural perturbation remains local, or in the SIDD model where cooperative behaviour occurs through the long-range communication of superhelical stress (which cannot be partitioned into bending or writhing), in this case co-operative transitions are observed due to the bending stress within the closed circular topology. For the example of the doubly-kinked minicircles shown in Figure 3A, the comparative lack of bending stress in the two roughly equal length duplex sections either side of the kinks is clear, whereas the singly-kinked minicircles still have a region of high duplex curvature opposite the kink. This phenomenon of cooperative kink formation due to bending in small DNA loops has also been seen experimentally with cryo-EM (31), although the sequence dependence of this phenomenon could not be determined. Simulation studies of the DNA loop stabilizing protein HU have demonstrated a cooperative uptake of proteins that introduce sharp kinks in DNA at antipodal sites along a closed DNA circle, highlighting the critical role played by DNA topology in inducing site-specific recognition by otherwise non-specific DNA binding proteins (43).

### Structures of DNA defects from oxDNA and atomistic MD simulations

Bending stress and negative supercoiling both weaken the stacking interactions between consecutive DNA base pairs within the duplex. Representative examples of the global structures of denatured DNA minicircles at Δ*Lk* ≈ −1 in the oxDNA and atomistic MD simulations are shown in Figure 3A. We observe a rich variety of structural defects forming within the DNA circles in both the atomistic and coarse-grained models, as shown in Figure 3B. While many base pair melting and repair events can be sampled in a single 30 μs trajectory at the coarse-grained level, large structural disruptions such as kinks and denaturation bubbles are irreversible over the time scale accessible to the atomistic calculations due to the large entropic barrier associated with reforming a base pair after the formation of a defect, and so these simulations were continued up until a structural defect persisting for longer than 10ns was observed (see Figure 3C and Supplementary Table S2). The atomistic simulations consistently predict that irreversible denaturation events occur at pyrimidine-purine steps (YpR: TpA, CpA, and CpG) which have been shown to be particularly flexible by previous simulation studies (20,44) and by an analysis of the protein crystallographic database (45). An exception is the two base-pair slipping events in the poly-AA region of the DESIGNED sequence, which demonstrates the anomalous mechanical properties of repetitive DNA sequences. X-ray crystallography studies on linear A-tract DNA have shown that these sequences form bifurcated hydrogen bonds across the major groove, which are implicated in their unusual structural and mechanical behaviour (46). Type 1 kinks (in which all hydrogen bonding interaction between complementary base pairs remain intact but the stacking interactions are broken), type II kinks and denaturation bubbles (in which two or more successive complementary base pairs have broken) are observed with both simulation techniques. Wrinkled DNA structures appear transiently in the atomistic simulations, prior to the formation of denaturation bubbles. The DESIGNED sequence, which contains repetitive elements, was uniquely observed to relieve torsional stress through slip defects, which are implicated in genetic diversity in prokaryotes and diseases such as cancer in humans (47). Most strikingly, both the atomistic and coarse-grained DNA simulations show ‘cooperative kinking’, in which defects preferentially occur at opposite sides of the circle due to DNA bending. The choreography of an example of a co-operative denaturation event during the atomistic MD, and the corresponding energetic relaxation of the remaining sequence, is shown in Figure 3C.

### DNA base breathing in supercoiled DNA during atomistic MD simulations

**Figure 4.**
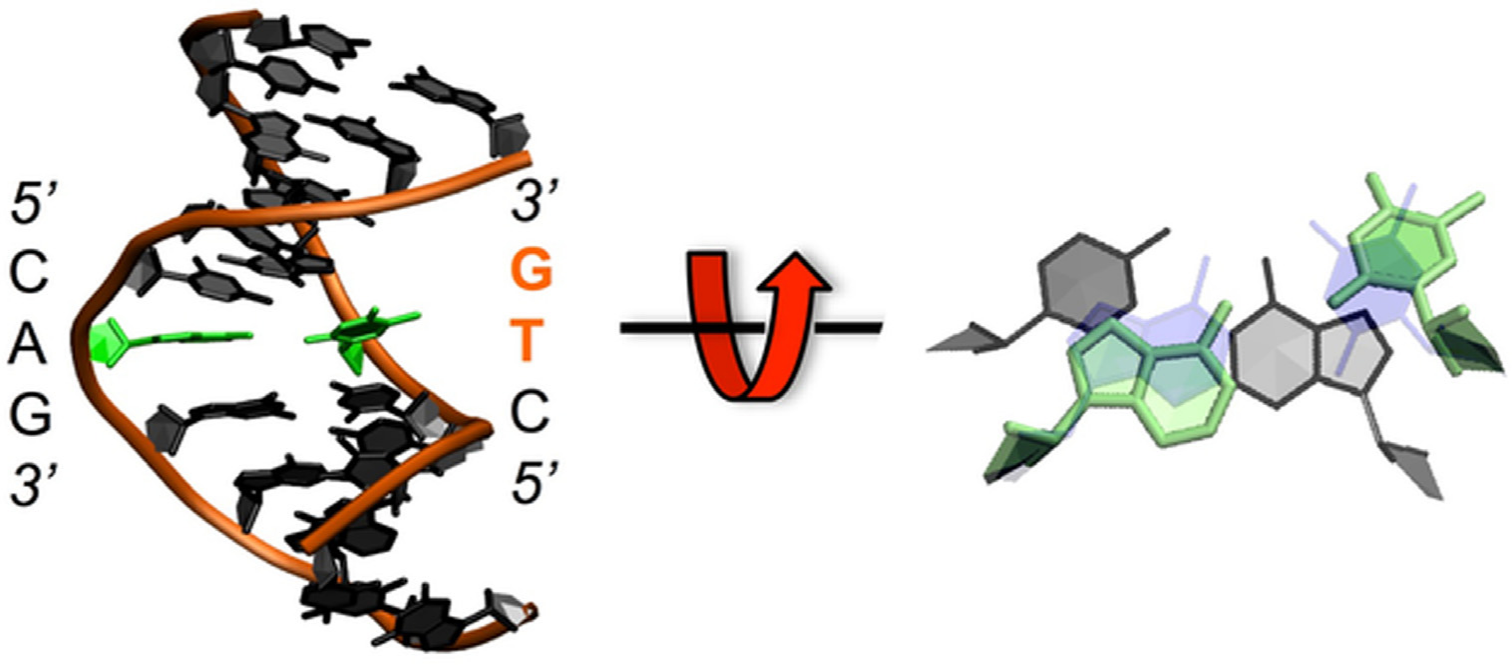
A representative example of DNA breathing from the atomistic MD simulations at Δ*Lk* ≈ −1 showing a thymine base pair flipped out into the major groove (in bold) relative to a stacked base pair (in shadow).

At the lower torsional stress of Δ*Lk* ≈ −0.5 (where the DNA is underwound by half a turn), the oxDNA and SIDD show similar sequence-dependent denaturation profiles as for Δ*Lk* ≈ −1 (see Supplementary Figure S2), however, the oxDNA simulations show that the average denaturation probability is reduced by a factor of 1.6 for each of the small circles (although intuitively a greater reduction might be expected, the bending stress makes a major contribution in these very small circles and is virtually unchanged). While no irreversible (>10ns) denaturation events are observed in the atomistic MD, multiple reversible breathing events in which a single DNA base flips out from the duplex stack are observed (see Table 1). Figure 4 shows a sample breathing structure at a CAG trinucleotide, in which the central thymine base has flipped into the major groove. More than 85% of breathing events in all the simulations performed at Δ*Lk* ≈ -0.5 involved CA, TA, CG or AT base pair steps. This is in contrast to the behaviour of the previous long time scale (μs) atomistic MD simulations on torsionally relaxed short linear DNA sequences, in which base pair fraying only occurs from the ends (48,49). Our calculations therefore show that negative supercoiling promotes ba*s*e-pair opening events. Supercoiling is ubiquitous in bacteria, and residual supercoiling may well persist in eukaryotic chromatin when the DNA is unwrapped from histones and subjected to the torsional forces associated with transcription or replication (50). Therefore, the observation of a significant increase in base opening events in supercoiled DNA has implications for the plethora of biological processes that disrupt complementary base pairing, such as detection of damage by DNA repair enzymes, some of which have been shown to operate by flipping individual bases out into the active site (51) as well for bacterial transcription and DNA replication.

### Non-local sequence dependent minicircle mechanics in atomistic MD

The collective sequence-dependent structure and flexibility of each DNA sequence and the nature of the topological constraint give rise to highly specific properties for each minicircle in such a way that changing one half of the sequence (e.g. inserting the FUSE element in place of a section of RANDOM DNA) affects the mechanics of the entire minicircle, not just the region that has been perturbed. The degree of destabilization of the duplex due to supercoiling depends on the superhelical density, which contains a hidden sequence dependence due to the inherent variability of the relaxed twist of each base step, which is significant for circles that are very small. Counter-intuitively, the highest base breathing rate for Δ*Lk* ≈ −0.5 in the atomistic MD simulations occurred in the DESIGNED sequence, and significantly less was observed in the AT-rich FUSE embedded minicircle (see Table 1) compared to the other two sequences. Moreover, the irreversible denaturation events at Δ*Lk* ≈ −1 consistently occurred after shorter amounts of atomistic MD in the RANDOM compared to the FUSE sequence, in spite of the higher AT content of the FUSE-embedded circle (see Supplementary Table S2). This initially unexpected observation is due to both the specific sequence-dependent twist and flexibility of the three minicircles at the atomistic level. Similarly, counterintuitive sequence-dependent behaviour was observed by single molecule manipulation experiments to investigate supercoiling induced melting, and, as here, was ascribed to the delocalized effect of bubble formation combined with subtle sequence-dependent changes in bending and twisting rigidity associated with plectoneme formation (52).

The equilibrium twist of each base pair step in the atomistic MD simulations is parameterized to mimic the sequence dependence observed in the X-ray and NMR structures available in the Protein Data Bank (53), which can be as low 29° per bp (for AG steps), but as high as 42° per bp (for GA) (54). Consequently, the superhelical density of a minicircle can also be highly sequence dependent. For example, in the experiments by Du *et al*. an 84 bp minicircle gave two topoisomers on cyclization, even though this circle should have zero linking number difference for a twist of 10.5 bp per helical turn (22). A calculation of the relaxed twist for each of the three sequences studied here (see Table 1) from a series of atomistic MD simulations on linear DNA fragments (see Materials and Methods) shows that while Δ*Lk* ≈ −0.5 for all of the circles, the DESIGNED sequence was actually subjected to significantly higher levels of negative superhelical stress as a result of its anomalously high helical twist, which promotes DNA breathing. It is only in the limit of infinitely long plasmids with a sufficiently heterogeneous sequence that the sequence dependence of the DNA twist becomes negligible; three small minicircles of the same size and linking number but with different sequences are likely to have significantly different superhelical densities.

Analysis of the protein-DNA complexes in the Protein Data Bank (45,53) and long timescale atomistic MD calculations (55) have shown that DNA flexibility is also highly sequence dependent, with TA and CA steps observed to be the most flexible (54). To estimate the elastic energy stored within each of the three circles within the harmonic approximation, which enables a simple comparison between the mechanical energy stored in the three sequences, we constructed the stiffness matrix for each base pair step using the average values of helical parameters from a series of atomistic MD calculations of the appropriate linear sequences (see Supplementary Information and Supplementary Table S4) (41). We then compared the elastic stress present in the time-averaged structures of each minicircle sequence at Δ*Lk* ≈ −0.5 (see Table 1). The elastic energy stored is the lowest for the FUSE-embedded minicircles, predominantly due to the low twist energy (see Supplementary Information Table S6). The FUSE sequence has the highest content of flexible TA and CA steps, and is therefore most able to absorb superhelical stress through bending and writhing. The increased deformability of the FUSE-containing sequence implies that this minicircle is more mechanically robust when subjected to a given superhelical stress during the atomistic MD relative to the RANDOM sequence because lower mechanical stress within the intact state increases the free energy barrier between the intact and denatured states. This effect is not captured by the oxDNA and SIDD models, as these are parameterized relying primarily on thermodynamic information without sequence dependent mechanics.

## DISCUSSION

We have employed three complementary modelling methods at different levels of resolution to explore the sequence dependence of denaturation in small DNA loops under topological stress. As each of these theoretical approaches provides complementary information, the greatest insight is obtained by applying the three techniques synergistically to explore the sequence dependence of supercoiling dependent DNA denaturation. These diverse approaches all show that the propensity of an individual base pair to bend, to kink or open is a function not only of its own identity and the neighbouring base steps, as is generally the case for linear DNA (56), but is an emergent property determined by a subtle interplay of non-local effects.

The superhelical stress, which is determined by the linking number difference Δ*Lk*, is a global quantity for a topologically closed system. It induces mechanical stress which is relieved when a kink or bubble forms at a susceptible site, which then reduces the likelihood of subsequent defect formation throughout the rest of the circle. Therefore, within a restrained DNA topology it is possible to suppress denaturation at a given position by introducing a less mechanically robust sequence at a distal site, which will act as a sink for the superhelical stress, as shown by oxDNA and SIDD when the AT-rich FUSE element replaced one half of the RANDOM sequence circle. The relaxed linking number is a global property that depends upon the twist of each individual base step within the sequence. In the atomistic MD, the sequence dependence of the global twist was sufficiently large that the GC-rich DESIGNED minicircle unexpectedly had higher base breathing rates than the equivalent AT-rich topoisomer due in part to its higher superhelical density (see Table 1). While in large plasmids these sequence dependent effects are generally averaged out, in repetitive sequences large deviations can be additive if the repeated motif differs significantly in twist from the mean, which can give rise to unexpected mechanical properties in closed DNA topologies. The overall response of the DNA to superhelical stress is additionally dependent on the relative arrangements of particular sequence motifs within the minicircle. To relieve the maximum bending stress, kink defects occurred co-operatively at opposite sides of the minicircles in both the oxDNA and atomistic simulations. In the atomistic MD, placing flexible elements at opposite sides of the circle facilitated global DNA bending in the AT-rich FUSE sequence, which affected the partitioning between twist (torsional stress) and writhe (bending stress). An equivalent physical mechanism whereby bending flexibility affects the stability of plectonemic DNA through its influence on the twist-writhe partition has been identified by single molecule studies (52). Increasing GC content was found to promote DNA melting, because the higher bending rigidity of the GC-rich DNA suppressed the release of superhelical stress through writhing (52). This subtle interplay between DNA bending, writhing and denaturation is controlled by the mechanical properties of the entire DNA sequence in these restrained DNA topologies.

While methods to efficiently determine the chemical sequence of the DNA bases in a genome, including epigenetic modifications, are now mature, and both the transcriptome and the corresponding proteome can be quantified, further experimental developments are required to probe how the physical status of the DNA contributes to regulation. In the model organism *E. coli*, a comparative genomics analysis suggested that genes are arranged on the bacterial chromosome so as to optimize the coupling of temporal gene expression patterns within neighbouring genes, where the levels of supercoiling are regulated locally within a domain at any one time through transcription-coupled DNA supercoiling (57). Such a direct physical mechanism reduces the number of transcription binding sites required to impose genomic control and offers a potentially faster and more efficient mechanism for modulating gene expression than interactions with transcription factor binding interactions (57). In human lymphoma cells, measurements of the superhelical density associated with transcription suggests a value of ∼ -0.07 extending around 1 to 1.5 kB from the polymerase enzyme (5), which according to the calculations here is sufficient to induce structural disruptions within the duplex structure. Using a novel protocol to map ssDNA across whole genomes in lymphocytes, Kouzine *et al* demonstrated that the melting of promotors provides a key regulatory step in global gene expression, and in this case enables a rapid response of the immune system to invading pathogens (58). Our understanding of how physical signals, such as supercoiling, can communicate regulatory information has lagged behind that of protein and RNA based gene control mechanisms, simply because the transient nature of stress mediated inter-gene communication is more difficult to observe and quantify experimentally. However, the universality of genome compartmentalization suggests that topologically closed synthetic plasmids containing the appropriate biologically active elements and sites for structural and regulatory DNA binding proteins might well provide sufficiently sophisticated model systems to mimic *in vivo* processes, and facilitate the study of the importance of delocalized DNA mechanics both theoretically and experimentally.

## Supplementary Material

Supplementary Data
