# Supplementary material for: Long-range correlations in the mechanics of small DNA circles under topological stress revealed by multi-scale simulation
Source: Nucleic Acids Res. 2016 Sep 22;44(19):9121–30. doi: 10.1093/nar/gkw815 (PMC5100592; doi:10.1093/nar/gkw815)
Supplement: Supplementary Data [file gkw815_Supp.docx]

**Supplementary Data**

**Long-range correlation in the mechanics of small DNA circles under topological stress revealed by multi-scale simulation**

Thana Sutthibutpong, Christian Matek, Craig Benham, Gabriel Gouvea-Slade, Agnes Noy, Charlie Laughton, Jonathan Doye, Ard Louis, and Sarah Harris

**1) Sequence Information**

**MINICIRCLES 100 bp for oxDNA simulations at** **superhelical density σ ≈ -0.05 (9 turns)**

RANDOM sequence: Sequence was from Du et al (Du et al. 2008).

TTGGCAGTTA ATCGAACAAG ACCCGTGCAA TGCTATCGAC ATCAAGGCCT ATCGTTACGG GGTTGGGAGT CAATGGGTTC AGGATGCAGG TGAGGATATC

FUSE-embedded sequence: **FUSE element (in BOLD)** is found in the human c-myc gene (Kouzine et al. 2004; Kouzine et al. 2008) and replaces half of the RANDOM sequence.

**TATATTTAAT ATATAATGTA TATTCCCTCG GGATTTTTTA TTTTGTGTTA TT**TATCGCTA TTACGGGGTT GGGAGTCAAT GGGTTCAGGA TGCAGGTATC

DESIGNED sequence:

^1^CGCGCGCGCGCGC **^14^TATATATATATA** ^26^CGCGCGCGCGCGC **^39^ATATATATATAT** ^51^CGCGCGCGCGCGC **^64^AAAAAAAAAAAA** ^76^CGCGCGCGCGCGC **^89^CACACACACACA**

**MINICIRCLES 106 bp for oxDNA simulations at** **superhelical density σ ≈ -0.1 (9 turns)**

RANDOM sequence: Sequence was from Du et al (Du et al. 2008)

TTTGCGGCAG TTAATCGAAC AAGACCCGTG CAATGCTATC GACATCAAGG CCTATCGCTA TTACGGGGTT GGGAGTCAAT GGGTTCAGGA TGCAGGTGAG GATATC

FUSE-embedded sequence:

**TATATTTAAT ATATAATGTA TATTCCCTCG GGATTTTTTA TTTTGTGTTA TT**TATCGCTA TTACGGGGTT GGGAGTCAAT GGGTTCAGGA TGCAGGTGAG GATATC

DESIGNED sequence:

^1^CGCGCGCGCGCGC **^14^TATATATATATA** ^26^CGCGCGCGCGCGCGC **^41^ATATATATATAT** ^53^CGCGCGCGCGCGCGC **^68^AAAAAAAAAAAA** ^80^CGCGCGCGCGCGCGC **^95^CACACACACACA**

**MINICIRCLES 102 bp for Atomistic MD simulations at** **superhelical density σ ≈ -0.05 (9 turns)**

RANDOM sequence:

TTGCGGCAGT TAATCGAACA AGACCCGTGC AATGCTATCG ACATCAAGGC CTATCGTATT ACGGGGTTGG GAGTCAATGG GTTCAGGATG CAGGTGAGGA TC

FUSE-embedded sequence:

**TATATTTAAT ATATAATGTA TATTCCCTCG GGATTTTTTA TTTTGTGTTA TT**TATCGCTA TTACGGGGTT GGGAGTCAAT GGGTTCAGGA TGCAGGTGAA TC

DESIGNED sequence:

^1^CGCGCGCGCGCGCGC ^16^**TATATATATATA** ^28^CGCGCGCGCGCGC **^41^ATATATATATAT** ^53^CGCGCGCGCGCGC **^66^AAAAAAAAAAAA** ^78^CGCGCGCGCGCGC **^91^CACACACACACA**

**MINICIRCLES 108 bp for Atomistic MD simulations at** **superhelical density σ ≈ -0.1 (9turns)**

RANDOM sequence:

TTTGCGGCAG TTAATCGAAC AAGACCCGTG CAATGCTATC GACATCAAGG CCTATCGCTA TTACGGGGTT GGGAGTCAAT GGGTTCAGGA TGCAGGTGAG GATATATC

FUSE-embedded sequence:

**TATATTTAAT ATATAATGTA TATTCCCTCG GGATTTTTTA TTTTGTGTTA TT**TATCGCTA TTACGGGGTT GGGAGTCAAT GGGTTCAGGA TGCAGGTGAG GATATATC

DESIGNED sequence:

^1^CGCGCGCGCGCGCGC **^16^TATATATATATA** ^28^CGCGCGCGCGCGCGC **^43^ATATATATATAT** ^55^CGCGCGCGCGCGCGC **^70^AAAAAAAAAAAA** ^82^CGCGCGCGCGCGCGC **^97^CACACACACACA**

**LINEAR 43 bp oligomers**

Sequence 1: GCGCGCAAATATAAGTACATATTGATCTGGAGCTCGACGCGCG

Sequence 2: GCGCGCTAAAATTAGAACTTAATGTTCAGGTGCACGTCGCGCG

Sequence 3: GCGCGCGAACATGAGCACGTACTGGTCCGGGGCCCGGCGCGCG

Sequence 4: GCGCGCCAAGATCAGGACCTAGTGCTCGGGCGCGCGCCGCGCG

Sequence 5: CGCGCGCGCAAAAATAAGAACATAATTATGATCACGCGCGCGC

Sequence 6: CGCGCGCGCAGAAGTAGGAGCACAACTACGACCACGCGCGCGC

Sequence 7: CGCGCGCGCGAAGATGAGGACGTAGTTGTGGTCGCGCGCGCGC

Sequence 8: CGCGCGCGCGGAGGTGGGGGCGCAGCTGCGGCCGCGCGCGCGC

Sequence 9: CGCGCGCGCAAAGATAAGGGTATAGAAATGGGAACGCGCGCGC

Sequence 10: CGCGCGCGCAGAGACAGGGGCACAGAGACGGGGACGCGCGCGC

Sequence 11: CGCGCGCGCGTTAATGCTAACGTAATTGCAATCGCGCGCGCGC

Sequence 12: CGCGCGCGCGTCAGTGCCAGCGTGACTGCGACCGCGCGCGCGC

Sequence 13: CGCGCGCGCAAACATAAGCACATACTTATGCTCACGCGCGCGC

Sequence 14: CGCGCGCGCAGACGTAGGCGCACACCTACGCCCACGCGCGCGC

Sequence 15: CGCGCGCGCCAAAATCAGAACCTAATTCTGATCCCGCGCGCGC

Sequence 16: CGCGCGCGCCGAAGTCGGAGCCCGACTCCGACCCCGCGCGCGC

**2) Detailed Simulation Protocols for atomistic MD Simulations**

Multistate minimisation and equilibration protocols were taken from our previous work by (Mitchell et al. 2011), which was adapted from the standard protocol in the study of DNA triplexes (Shields et al. 1997). Table S1 shows all the step of minimization and equilibration protocol to prepare the starting structures for the production MD runs. Production runs were at a constant temperature (300K) and pressure (1 atm), with the Berendsen weak coupling scheme and the volume rescaled. The Particle Mesh Ewald (PME) electrostatic cutoff was set to be 9.0 Å. The SHAKE algorithm was applied to the covalent bonds including hydrogen and a simulation timestep of 2 fs was used, and the molecular trajectory was recorded every 1 ps. Production MD simulations were run using the GROMACS 4.5 program on the University of Leeds supercomputers ARC1, ARC2, the N8 HPC supercomputer, POLARIS and the XSEDE Stampede supercomputing resource. A sufficiently detailed description of the minicircle set-up, simulation and analysis (including input files and scripts) for these simulations to be replicated is provided in our methodological publication (Sutthibuttpong, Noy & Harris 2016).

| **Equilibration Stage** | **All-atom DNA Restraints** |  |
| --- | --- | --- |
| **Mininisation 1** | k = 500.0 kCal/mol/Å^2^ | 10000 cycles |
| **Mininisation 2** | k = 50.0 kCal/mol/Å^2^ | 10000 cycles |
| **Mininisation 3** | k = 25.0 kCal/mol/Å^2^ | 10000 cycles |
| **Mininisation 4** | No restraints | 10000 cycles |
| **Equilibration 1** | k = 500.0 kCal/mol/Å^2^, T = 100K | 10 ps |
| **Equilibration 2** | k = 50.0 kCal/mol/Å^2^, T = 300K | 10 ps |
| **Equilibration 3** | k = 50.0 kCal/mol/Å^2^, T = 300K | 10 ps |
| **Equilibration 4** | k = 25.0 kCal/mol/Å^2^, T = 300K | 10 ps |
| **Equilibration 5** | k = 10.0 kCal/mol/Å^2^, T = 300K | 10 ps |
| **Equilibration 6** | k = 5.0 kCal/mol/Å^2^, T = 300K | 10 ps |
| **Equilibration 7** | k = 2.5 kCal/mol/Å^2^, T = 300K | 10 ps |
| **Equilibration 8** | k = 1.0 kCal/mol/Å^2^, T = 300K | 10 ps |

**Table S1:** Force constants and durations of the minimisation and equilibration steps

| 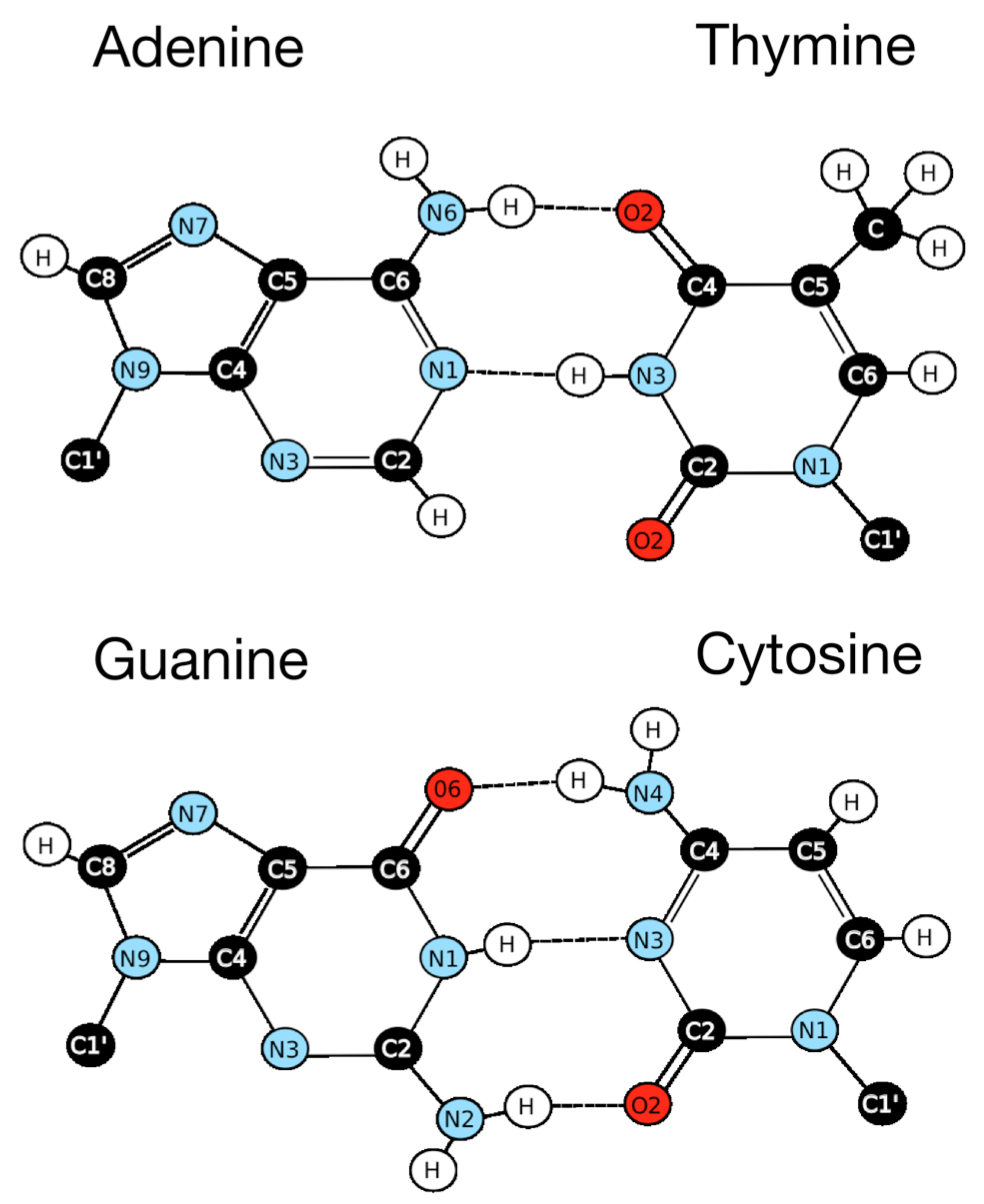 |
| --- |

**Figure S1:** Nomenclatures for the atoms in DNA nucleobases


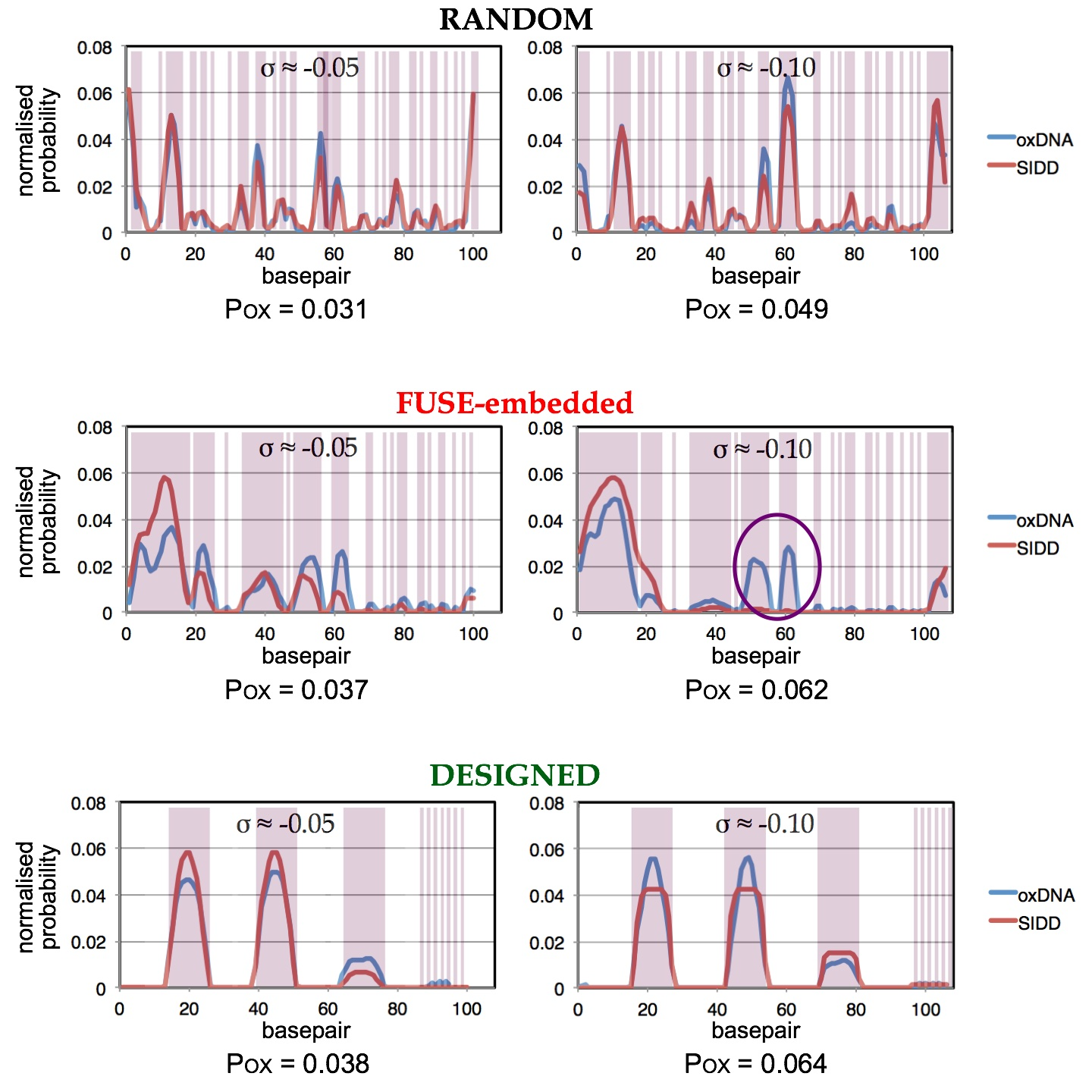


**Figure S2:** Normalised relative probability profiles calculated from RANDOM, FUSE-embedded and DESIGNED sequences by SIDD model (red) and an OxDNA simulation (blue). A-T base pair regions are shaded. The total denaturation probability calculated by oxDNA is shown below each plot. Secondary denaturation sites due to co-operative kinking in the oxDNA simulations of highly negative supercoiled 106 bp FUSE-embedded minicircles (as described in the Results section of the main manuscript) are circled. The atomistic simulations do not provide sufficient sampling for denaturation probabilities to be assigned, and so are not shown here.

| Name | Denaturation | Time (ns) | Sequence | location |
| --- | --- | --- | --- | --- |
| **RANDOM-A-r1**  **RANDOM-B-r0**  **RANDOM-B-r1**  **RANDOM-C-r1**  **RANDOM-A-r0**  **RANDOM-A-r2**  **RANDOM-B-r2**  **RANDOM-C-r0**  **RANDOM-C-r2**  **FUSE-A-r0**  **FUSE-A-r2**  **FUSE-B-r1**  **FUSE-C-r1**  **FUSE-A-r1**  **FUSE-B-r0**  **FUSE-B-r2**  **FUSE-C-r0**  **FUSE-C-r2** | Untwisting  Untwisting  Untwisting  Untwisting  Bending  Bending  Bending  Bending  Bending  Bending  Bending  Bending  Bending  Writhing  Writhing  Writhing  Writhing  Writhing | 23  17  10  10  28  52  1  22  1  4  2  9  9  93  61  89  47  26 | C*AA*T/A*TT*G  C*AG*G/C*CT*G  T*TA*C/G*TA*A  T*CA*A/T*TG*A  C*CG*T/A*CG*G  C*A*A/T*T*G  A*T*G/C*A*T  T*AT*T/A*AT*A  C*AA*G/C*TT*G  T*A*A/T*T*A  T*TT*G/C*AA*A  A*T*G/C*A*T  C*GG*G/C*CC*G  T*A*T/A*T*A  T*ATT*A/T*AAT*A  A*TA*T/A*TA*T  T*ATC*G/C*GAT*A  T*ATA*A/T*TAT*A | 32-33  87-88  61-62  77-78  27-28  32  80  60-61  47-48  15  43-44  80  65-66  4  60-62  12-13  54-56  13-15 |

**Table S2:** Summary of defect formation events in σ = -0.1 atomistic MD simulations of the RANDOM and FUSE-embedded DNA minicircles, (Name) indicates the DNA sequence – register angles – and number of replicas (r0, r1 and r2) for each simulation, (Denaturation) indicates the global origin of the stress driving defect formation; we have classified structural distortions as arising due to helical untwisting (bubbles), DNA bending (kinking) or writhing (which suppresses kink and bubble formation), (Time) the amount of time after starting each simulation when the first base pair disruption was observed, and (Sequence / location) indicates the position and base sequence where the denaturation started for each simulation.

| **NAME** | **Base pair**  **POSITION** | **Sequence** | **TIME**  **(ns)** |  |
| --- | --- | --- | --- | --- |
| **TA-Major_r1** | 57,58 | **CG** | 10 | 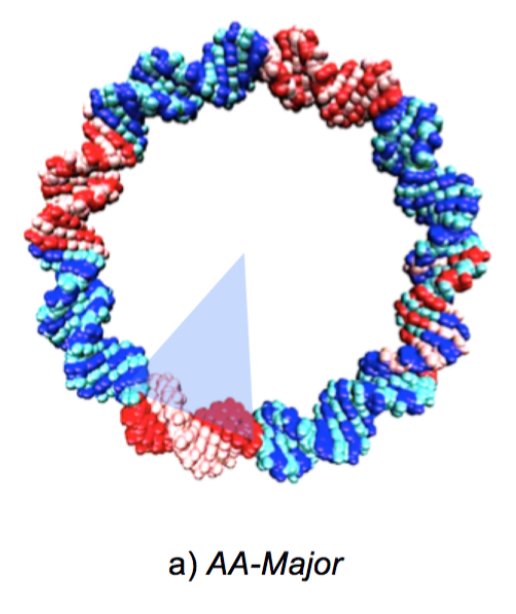  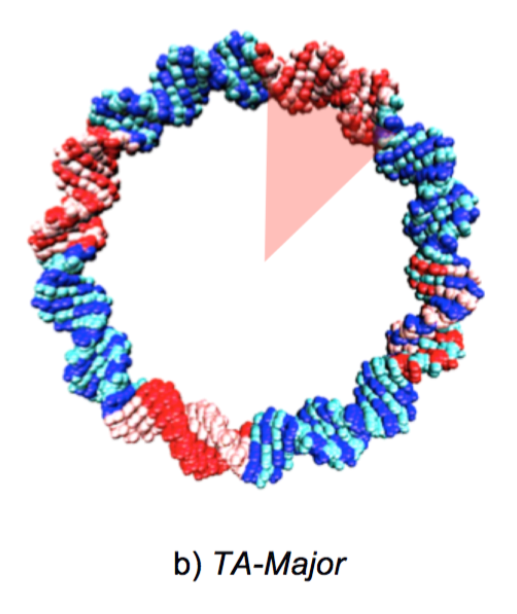  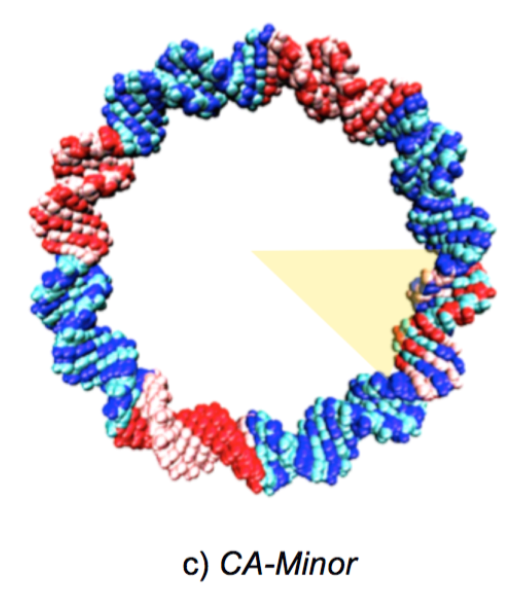  **TableS3:** Structural disruptions observed within the 45 simulations of the DESIGNED-sequence at σ = -0.1. Simulation names indicate the register angles (shown above) and the replica number. A description of the base pair positions for the defects, the DNA sequences and the time taken for the defect formation are provided. |
| **TA-Major_r2** | 68,69 | **CG** | 3 |  |
| **TA-Major_r3** | 100 | **CA** | 3 |  |
| **TA-Major_r4** | 7,8 | **CG** | 13 |  |
| **TA-Major_r5** | 77,78,79,80 | **AA** | 30 |  |
| **TA-Major_r6** | 80 | **AA** | 25 |  |
| **TA-Major_r7** | 50,51 | **AT** | 14 |  |
| **TA-Major_r8** | 8,9 / 68 | **CG / CG** | 16 9 |  |
| **TA-Major_r9** | 63,64 / 101 | **CG / CA** | 7, 13 |  |
| **TA-Major_r10** | 10 | **CG** | 29 |  |
| **TA-Major_r11** | 79,80,81,82 | **AA / CG** | 4 |  |
| **TA-Major_r12** | 9,10 | **CG** | 2 |  |
| **TA-Major_r13** | 108, 1 | **CA / CG** | 20 |  |
| **TA-Major_r14** | 92,93 | **CG / CA** | 8 |  |
| **TA-Major_r15** | 44-52 | **AT** | 14 |  |
|  |  |  |  |  |
| **AA-Major_r1** | 25 / 85 | **TA / CG** | 13, 7 |  |
| **AA-Major_r2** | 21,22 / 70-77 | **TA / AA** | 2 |  |
| **AA-Major_r3** | 65,66,67 | **CG** | 17 |  |
| **AA-Major_r4** | 106, 107 | **CA** | 3 |  |
| **AA-Major_r5** | 79,80,81 | **CG / AA** | 3 |  |
| **AA-Major_r6** | 55 | **CG** | 7 |  |
| **AA-Major_r7** | 19,20 | **TA** | 3 |  |
| **AA-Major_r8** | 84,85 | **CG** | 6 |  |
| **AA-Major_r9** | 26,27 | **TA** | 25 |  |
| **AA-Major_r10** | 79,80,81 | **CG / AA** | 3 |  |
| **AA-Major_r11** | 44,45,46 | **AT** | 5 |  |
| **AA-Major_r12** | 44 / 103, 104 | **AT / CA** | 21 |  |
| **AA-Major_r13** | 63,64 | **CG** | 18 |  |
| **AA-Major_r14** | 45 | **TA** | 37 |  |
| **AA-Major_r15** | 49-52 / 94,95 | **TA / CA** | 5, 10 |  |
|  |  |  |  |  |
| **CA-Minor_r1** | 30,31 | **CG** | 21 |  |
| **CA-Minor_r2** | 61,62,63 | **CG / AA** | 9 |  |
| **CA-Minor_r3** | 102, 103 | **CA** | 17 |  |
| **CA-Minor_r4** | 35,36,37 | **CG** | 42 |  |
| **CA-Minor_r5** | 25,26,27 | **AT** | 12 |  |
| **CA-Minor_r6** | 18,19,20 | **CA** | 37 |  |
| **CA-Minor_r7** | 104 | **CA** | 23 |  |
| **CA-Minor_r8** | 106, 107, 108 | **CA** | 12 |  |
| **CA-Minor_r9** | 36,37 / 85 | **CG / CG** | 13 |  |
| **CA-Minor_r10** | 99, 100, 101 | **CA** | 2 |  |
| **CA-Minor_r11** | 98,99,100,101 | **CA** | 10 |  |
| **CA-Minor_r12** | 87 | **CG** | 10 |  |
| **CA-Minor_r13** | 45,46/105,106 | **AT / CA** | 29, 8 |  |
| **CA-Minor_r14** | 86 87 | **CG** | 20 |  |
| **CA-Minor_r15** | 61,62,63 | **CG** | 2 |  |

|  | **Shift (Å)** | | | **Slide (Å)** | | | **Rise (Å)** | | | **Tilt (°)** | | | **Roll (°)** | | | **Twist (°)** | | |
| --- | --- | --- | --- | --- | --- | --- | --- | --- | --- | --- | --- | --- | --- | --- | --- | --- | --- | --- |
| **TA** | 0.13 | ± | 0.91 | -0.04 | ± | 0.81 | 3.26 | ± | 0.36 | 0.49 | ± | 5.04 | 9.81 | ± | 7.53 | 31.4 | ± | 6.8 |
| **CA / TG** | 0.10 | ± | 0.77 | -0.22 | ± | 0.63 | 3.25 | ± | 0.39 | 0.42 | ± | 4.73 | 10.80 | ± | 6.50 | 29.7 | ± | 7.5 |
| **CG** | 0.04 | ± | 0.88 | 0.00 | ± | 0.61 | 3.19 | ± | 0.38 | 0.61 | ± | 5.31 | 8.98 | ± | 6.76 | 31.1 | ± | 8.4 |
| **AA / TT** | 0.06 | ± | 0.73 | -0.24 | ± | 0.59 | 3.31 | ± | 0.29 | -0.37 | ± | 4.74 | 2.13 | ± | 5.80 | 34.7 | ± | 5.5 |
| **AG / CT** | 0.05 | ± | 0.86 | -0.52 | ± | 0.66 | 3.39 | ± | 0.33 | -0.19 | ± | 4.81 | 3.62 | ± | 5.65 | 33.3 | ± | 5.8 |
| **GA / TC** | 0.14 | ± | 0.80 | -0.04 | ± | 0.71 | 3.35 | ± | 0.31 | 0.79 | ± | 4.79 | 2.27 | ± | 5.97 | 36.1 | ± | 5.8 |
| **GG / CC** | 0.03 | ± | 0.76 | 0.65 | ± | 0.82 | 3.51 | ± | 0.36 | 0.45 | ± | 4.49 | 4.63 | ± | 5.57 | 33.2 | ± | 5.7 |
| **AT** | 0.00 | ± | 0.66 | -0.74 | ± | 0.39 | 3.21 | ± | 0.27 | 0.18 | ± | 3.79 | -0.16 | ± | 4.99 | 30.5 | ± | 4.0 |
| **AC / GT** | 0.11 | ± | 0.74 | -0.66 | ± | 0.46 | 3.31 | ± | 0.29 | 0.12 | ± | 3.91 | -0.07 | ± | 5.66 | 31.6 | ± | 4.6 |
| **GC** | -0.01 | ± | 0.75 | -0.48 | ± | 0.53 | 3.38 | ± | 0.28 | 0.18 | ± | 4.21 | -0.34 | ± | 6.27 | 34.2 | ± | 4.9 |
| **Average** | **0.07** | **±** | **0.78** | **-0.21** | **±** | **0.63** | **3.33** | **±** | **0.33** | **0.24** | **±** | **4.58** | **4.07** | **±** | **5.99** | **32.8** | **±** | **5.9** |

**TableS4:** Averages and standard deviations of the six base pair step parameters at ten different di-nucleotide steps calculated from a series of 43 bp linear DNA simulations. Standard deviations (SD) are shaded: darker shading corresponds to higher SD values and greater flexibility.

| **Elastic Constants from Diagonal Elements** | | | | | | |
| --- | --- | --- | --- | --- | --- | --- |
|  | **K**shift | **K**slide | **K**rise | **K**tilt | **K**roll | **K**twist |
|  | [ kBT deg^-2^ ] | [ kBT deg^-2^ ] | [ kBT deg^-2^ ] | [ kBT Å^-2^ ] | [ kBT Å^-2^ ] | [ kBT Å^-2^ ] |
| **TA** | 1.107 | 2.213 | 11.127 | 0.041 | 0.030 | 0.041 |
| **CA / TG** | 1.683 | 3.089 | 10.545 | 0.046 | 0.030 | 0.034 |
| **CG** | 1.997 | 3.548 | 10.400 | 0.052 | 0.027 | 0.029 |
| **AA / TT** | 2.147 | 4.652 | 12.665 | 0.046 | 0.036 | 0.057 |
| **AG / CT** | 1.545 | 3.683 | 11.644 | 0.051 | 0.034 | 0.046 |
| **GA / TC** | 1.659 | 3.489 | 12.306 | 0.054 | 0.037 | 0.059 |
| **GG / CC** | 1.840 | 2.667 | 11.747 | 0.062 | 0.034 | 0.052 |
| **AT** | 2.468 | 8.536 | 16.875 | 0.071 | 0.049 | 0.079 |
| **AC / GT** | 1.825 | 6.073 | 16.044 | 0.066 | 0.039 | 0.064 |
| **GC** | 2.417 | 4.863 | 16.787 | 0.069 | 0.037 | 0.061 |
|  |  |  |  |  |  |  |
| **Elastic Constants from Inverse Variances** | | | | | | |
|  | **K**shift | **K**slide | **K**rise | **K**tilt | **K**roll | **K**twist |
|  | [ kBT deg^-2^ ] | [ kBT deg^-2^ ] | [ kBT deg^-2^ ] | [ kBT Å^-2^ ] | [ kBT Å^-2^ ] | [ kBT Å^-2^ ] |
| **TA** | 1.208 | 1.524 | 7.716 | 0.039 | 0.018 | 0.022 |
| **CA / TG** | 1.687 | 2.520 | 6.575 | 0.045 | 0.024 | 0.018 |
| **CG** | 1.291 | 2.687 | 6.925 | 0.035 | 0.022 | 0.014 |
| **AA / TT** | 1.877 | 2.873 | 11.891 | 0.045 | 0.030 | 0.033 |
| **AG / CT** | 1.352 | 2.296 | 9.183 | 0.043 | 0.031 | 0.030 |
| **GA / TC** | 1.563 | 1.984 | 10.406 | 0.044 | 0.028 | 0.030 |
| **GG / CC** | 1.731 | 1.487 | 7.716 | 0.050 | 0.032 | 0.031 |
| **AT** | 2.296 | 6.575 | 13.717 | 0.070 | 0.040 | 0.063 |
| **AC / GT** | 1.826 | 4.726 | 11.891 | 0.065 | 0.031 | 0.047 |
| **GC** | 1.778 | 3.560 | 12.755 | 0.056 | 0.025 | 0.042 |

**TableS5**: Elastic constants for the six base pair step parameters of the ten different di-nucleotide steps calculated from the diagonal terms of stiffness matrices (top) or from the variance (Noy et al. 2004) (bottom). Dark shading corresponds to low force constant and high flexibility. Elastic constants follow the same trends observed in other MD studies (Perez et al. 2008; Ivani et al. 2015), with pyrimidine-purine (YpR) being the most flexible steps (especially TA and CA) and purine-pyrimidine (RpY) the most rigid. While these values are approximate as they are derived from comparatively short simulations, our aim was to evaluate the relative (not absolute) elasticity for the three sequences and to rank them accordingly.

|  | **E***shift*  (k_B_T) | | | **E***slide*  (k_B_T) | | | **E***rise*  (k_B_T) | | | **E***tilt*  (k_B_T) | | | **E***roll*  (k_B_T) | | | **E***twist*  (k_B_T) | | | **E***total*  (k_B_T) | | | **Breathing**  **/ns** | | |
| --- | --- | --- | --- | --- | --- | --- | --- | --- | --- | --- | --- | --- | --- | --- | --- | --- | --- | --- | --- | --- | --- | --- | --- | --- |
| ***RANDOM*** | 1.11 | ± | 0.14 | 1.51 | ± | 0.17 | 1.14 | ± | 0.16 | 1.07 | ± | 0.14 | 1.38 | ± | 0.17 | 1.60 | ± | 0.17 | 7.80 | ± | 0.39 | 0.28 | ± | 0.03 |
| ***FUSE****-embed* | 1.03 | ± | 0.13 | 1.35 | ± | 0.17 | 1.05 | ± | 0.15 | 1.03 | ± | 0.13 | 1.36 | ± | 0.17 | 1.43 | ± | 0.16 | 7.24 | ± | 0.37 | 0.22 | ± | 0.03 |
| ***DESIGNED*** | 1.02 | ± | 0.15 | 1.48 | ± | 0.17 | 1.26 | ± | 0.16 | 1.00 | ± | 0.14 | 1.50 | ± | 0.17 | 1.59 | ± | 0.17 | 7.83 | ± | 0.40 | 0.32 | ± | 0.04 |

**TableS6:** Time-average of mechanical stress (in k_B_T) decomposed into base pair step rigid body parameters for each of the three DNA minicircles at ΔLk ≈ -0.5. Energy in each component was calculated using the elastic constants obtained from the diagonal elements of stiffness matrices in Table S5, the equilibrium parameters value from Table S4 and the rotational and translational displacements of a DNA base away from the average due to bending and supercoiling within the minicircle. Total stress corresponds to the breathing rates, as shown in Table 1 in the main text.

**SUPPLEMENTARY REFERENCES**

Du, Q., Kotlyar, A. & Vologodskii, A., 2008. Kinking the double helix by bending deformation. *Nucleic Acids Research*, **36**, 1120–1128.

Ivani, I. et al., 2015. Parmbsc1: a refined force field for DNA simulations. *Nature methods*, **13**, 55–58.

Kouzine, F. et al., 2004. The dynamic response of upstream DNA to transcription-generated torsional stress. *Nature structural & molecular biology*, **11**, 1092–1100.

Kouzine, F. et al., 2008. The functional response of upstream DNA to dynamic supercoiling in vivo. *Nature structural & molecular biology*, **15**, 146–154.

Mitchell, J.S., Laughton, C.A. & Harris, S.A., 2011. Atomistic simulations reveal bubbles, kinks and wrinkles in supercoiled DNA. *Nucleic Acids Research*, **39**, 3928–3938.

Noy, A. et al., 2004. Relative flexibility of DNA and RNA: A molecular dynamics study. *Journal of Molecular Biology*, **343**, 627–638.

Perez, A. et al., 2008. Towards a molecular dynamics consensus view of B-DNA flexibility. *Nucleic Acids Research*, **36** 2379–2394.

Shields, G.C., Laughton, C.A. & Orozco, M., 1997. Molecular Dynamics Simulations of the d(T·A·T) Triple Helix. *Journal of the American Chemical Society*, **119**, 7463–7469.

Sutthibutpong T., Noy A. & Harris S. A., 2016. Atomistic Molecular Dynamics Simulations of DNA Minicircle Topoisomers: A Practical Guide to Setup, Performance, and Analysis. In Chromosome Architecture: Methods and Protocols (edited by Mark C. Leake), Methods in Molecular Biology, **1431**, 196-219.
